# Clinical characteristics and annual incidence of pickleball-related upper extremity injuries among patients presenting to emergency departments, 2015-2024

**DOI:** 10.1016/j.jham.2026.100472

**Published:** 2026-05-27

**Authors:** Rishub K. Das, Wesley Thayer, Shady Elmaraghi, J. Bradford Hill, Brian C. Drolet

**Affiliations:** Department of Plastic Surgery, Vanderbilt University Medical Center, Nashville, TN, United States

**Keywords:** Elderly, Fracture, Hand, Pickleball, Upper extremity

## 1 Introduction

Participation in sports and recreational activities confers substantial health benefits, including improved cardiovascular fitness, mental well-being, and social engagement.^1^, ^2^, ^3^ However, these activities are also associated with a spectrum of injuries ranging from minor sprains and contusions to severe musculoskeletal trauma.^4^^,^^5^ Each year, approximately 2 million individuals in the United States experience sports-related injuries, contributing significantly to healthcare utilization and costs.^1^ Notably, the majority of acute fractures sustained during sports involve the upper extremity.^6^

Hand injuries account for approximately 20-30% of all emergency department (ED) visits in the United States.^7^ Surveillance of ED presentations for these injuries informs volume and resource planning, quality improvement, injury prevention, health equity, and postoperative care optimization. Pickleball—a hybrid paddle sport combining elements of tennis, badminton, and table tennis—was created in 1965 but has experienced an unprecedented rise in popularity over the past decade.^8^^,^^9^ Its accessibility, low cost, and suitability for outdoor play, particularly during the COVID-19 pandemic, have contributed to its widespread adoption across diverse age groups. As participation has expanded, hand and upper extremity surgeons are increasingly likely to encounter pickleball-related injuries.^8^^,^^10^ Yet, there is limited nationally representative data that characterizes the incidence and patterns of hand and upper extremity injuries among pickleball players. Understanding these trends may help guide practice management and public health initiatives to promote safe play and mitigate upper extremity morbidity. This study evaluates the clinical characteristics and annual estimated number of pickleball-related upper extremity injuries presenting to U.S. emergency departments (ED) between 2015 and 2024.

## 2 Materials and methods

We conducted a retrospective cross-sectional analysis of the National Electronic Injury Surveillance System (NEISS), a probability sample of hospitals in the United States that have an ED with data for every patient treated for an injury associated with consumer products. The database is sponsored by the Consumer Product Safety Commission and provides nationally representative data.^11^ The Vanderbilt University Institutional Review Board determined this study to be exempt from review and informed consent due to the de-identified nature of data collected. Collected information included patient demographic information such as age and sex, injury diagnosis, body part affected, and disposition. Each record includes a narrative text field that provides a short summary statement of the patient's history of present illness and mechanism of injury from ED documentation. Using the available documentation and data, each case is classified by mechanism of injury and associated consumer product or activity. We followed the Strengthening the Reporting of Observational Studies in Epidemiology (STROBE) reporting guideline.

Data from 2015 through 2024 were extracted from the NEISS and included if the injury affected the upper extremity defined as upper arm (shoulder, humerus), elbow, lower arm (forearm), wrist, hand, and finger. Pickleball-related injuries were identified using the narrative text field for cases that contained “pickleball” or “pickle ball.” Common misspellings including “pickel ball,” “pickl ball,” “pickelball,” and “picklball” were also searched to ensure no relevant cases were missed. Searches were not case sensitive. Injuries that did not affect the upper extremity were excluded (n = 9). Recurrent visits to the ED for the same patient may have been included as limited patient identifiers are provided to remove subsequent encounters. R.K.D. reviewed the included cases. Treatment year, age, sex, diagnosis, affected body part, and disposition were analyzed after appropriate case selection. Cases with multiple upper extremity injuries and where data were not available about the diagnosis (eg, fracture, laceration) were classified as “Other.”

All data analyses were performed using Stata 18.0 (StataCorp). Survey weights provided by the NEISS were used with Stata's *svy* framework to generate national estimates. Temporal trends were evaluated using weighted linear regression with year as a continuous predictor to estimate the annual increase in number of upper extremity injuries by year. This approach accounts for unequal variance across yearly estimates and yields efficient, unbiased estimates of annual change, consistent with the Gauss–Markov theorem. *P* < 0.05 was considered statistically significant. Data were analyzed from June 2025 to October 2025.

## 3 Results

Between 2015 and 2024, there were an estimated 30,906 (unweighted n = 544) pickleball-related upper extremity injuries with 6766 (21.9%, unweighted n = 123) occurring in 2024 (Table 1). Pickleball-related upper extremity injuries were more common among females (68.5%; 95% CI, 63.9%-72.7%) and patients 60 years or older (84.5%; 95% CI, 80.9%-87.6%). Most pickleball-related injuries affected the wrist (45%; 95% CI, 40.5%-49.8%) and were determined to be fractures (68.7%; 95% CI, 64.1%-72.8%). Among patients who presented to the ED with pickleball-related upper extremity injuries, 4.5% (95% CI, 3.0%-6.9%) required admission, 2.2% (95% CI, 1.1%-4.25) were transferred, and 93.1% (95% CI, 90.2%-95.1%) were treated and discharged without admission.

**Table 1:** Demographic characteristics, diagnoses, and Injured body parts among patients with pickleball-related upper extremity injuries, 2015-2024.

Variable	Unweighted No.	Weighted No. (%; 95% CI)^a^
Age, y		
0-18	17	488 (1.6; 0.1-3.4)
19-59	80	4291 (13.9; 10.2-17.9)
≥60	447	26,128 (84.5; 80.9-87.6)
Sex		
Male	169	9739 (31.5; 29.4-34.7)
Female	375	21,167 (68.5; 63.9-72.7)
Body Part		
Wrist	246	13,936 (45.1; 40.5-49.8)
Lower arm	92	5449 (17.6; 13.2-20.1)
Finger	54	2962 (9.6; 5.1-13.7)
Elbow	46	2527 (8.2; 4.2-11.3)
Upper arm	41	2255 (7.3; 3.1-10.9)
Hand	18	993 (3.2; 1.5-6.4)
Other^b^	47	2784 (9.0; 4.7-13.5)
Diagnosis		
Fracture	371	21,216 (68.7; 64.1-72.8)
Strain	52	2956 (9.6; 5.3-14.2)
Contusion	33	1754 (5.7; 2.1-9.5)
Laceration	25	1323 (4.3; 1.2-6.7)
Dislocation	17	1048 (3.4; 1.1-5.8)
Other^b^	46	2608 (8.4; 4.6-12.8)

a Percentages weighted using survey weights provided by the National Electronic Injury Surveillance System.

b Multiple injuries or data not available.

**Fig. 1:**
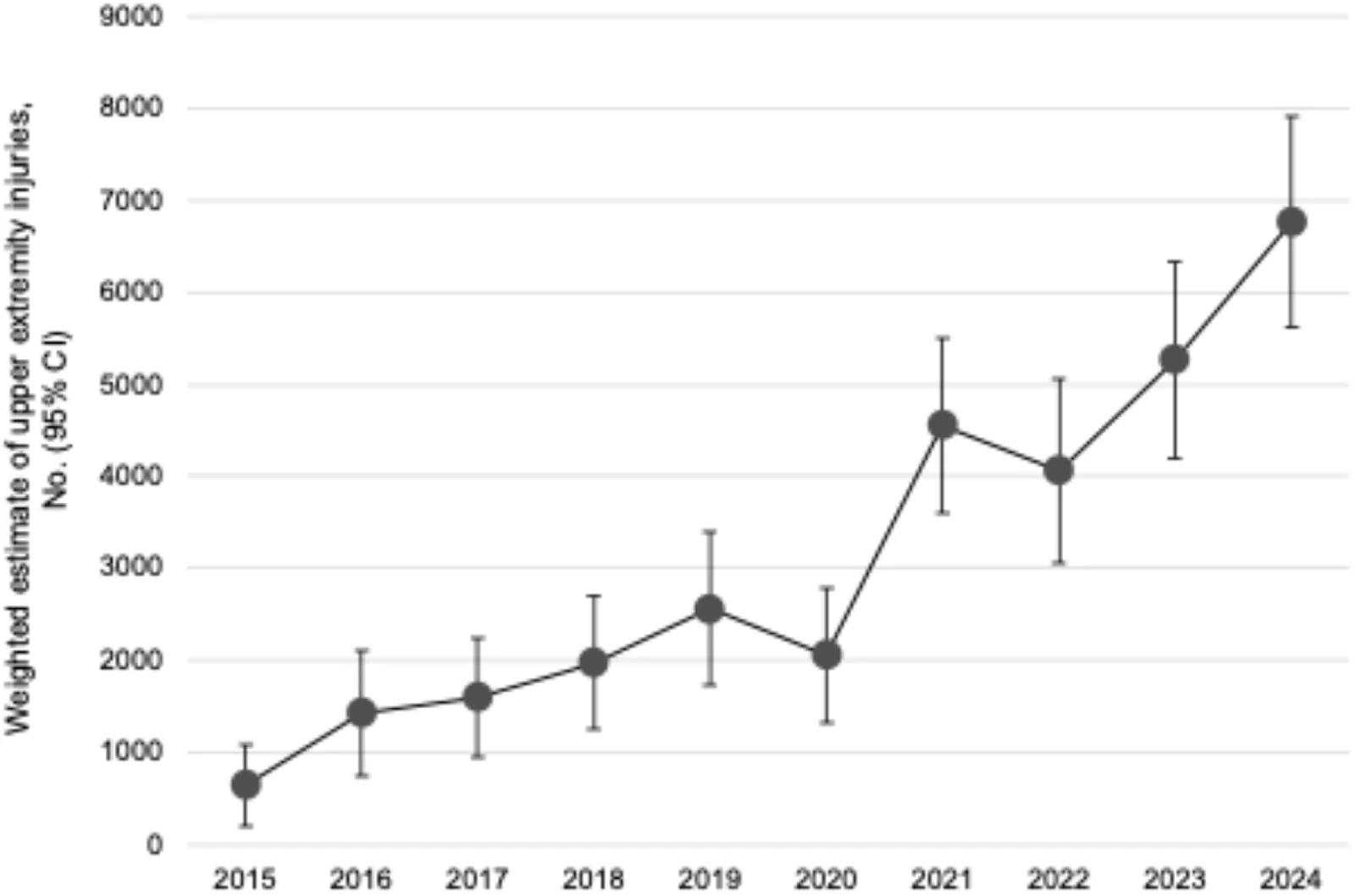
Weighted estimates of pickleball-related upper extremity injuries among patients presenting to the emergency department between 2015 and 2024.

Fig. 1 illustrates the number of pickleball-related upper extremity injuries by year. In 2015, there was an estimated 642 (95% CI, 203-1081; unweighted n = 9) compared with 6766 (95% CI, 5616–7917; unweighted n = 123) injuries in 2024, representing a growth of 953.9% (β = 615.5; 95% CI, 443.7–787.3; *P* < 0.001). Although the 2015 estimate was derived from a relatively small unweighted sample (n = 9), its influence on the overall trend was limited. Sensitivity analyses excluding 2015 yielded a similar slope estimate and did not meaningfully change the direction, magnitude, or statistical significance of the association, indicating that the regression results are stable and not driven by this single early data point.

## 4 Discussion

The number of pickleball-related upper extremity injuries has risen sharply over the past decade, particularly among older adults and women, with wrist fractures comprising the majority of cases. While most injuries were managed in the ED, a few cases required transfer or admission to the hospital for further care.

Pickleball-related injuries have risen over the past decade, outpacing participation growth according to national data.^12^ Participation in pickleball is estimated at 19.8 million players in the US and popularity has also grown globally including in South Korea.^12^^,^^13^ Across national studies, the most common pickleball-related injuries are soft tissue injuries in the knee and ankle, contrasting with the predominance of fractures reported in our study focused on upper extremity injuries.^14^ Men are more likely to sustain soft tissue injuries in the lower extremity, while women are more likely to sustain fractures and are more likely to fall.^14^^,^^15^

Our findings of increased fracture risk among elderly women aligns with established literature on sports-related trauma and osteoporosis.^16^, ^17^, ^18^ Women are at higher risk for fractures due to lower bone density, the effects of menopause on bone mineralization, and bone structure differences.^18^ Although physical activity provides substantial benefits in preserving mobility and preventing obesity, pickleball has significant injury risks that warrant targeted preventive strategies.^19^^,^^20^ Compared with traditional racquet sports such as tennis, pickleball involves shorter reaction times and closer player proximity, which may be associated with increased fall risk, particularly among individuals with balance or proprioceptive deficits. Promoting safe participation among at-risk populations through evidence-based guidelines may mitigate these injuries while maintaining the health benefits of the sport.^21^^,^^22^

One previous single-center retrospective study similarly highlighted the predominance of wrist injuries among pickleball players aligning with this study's nationally representative results.^8^ Annual trends were not described, but the authors report approximately 20% of patients required surgical treatment with an average time to surgery from initial injury of 88.5 days. Non-operative treatments included splinting, occupational therapy, and steroid injections.

Another study using self-reported data identified prolonged or frequent play—particularly sessions exceeding 2 h or occurring on consecutive days—as key risk factors for upper extremity injury, underscoring modifiable behavioral contributors to injury prevention especially among players older than 60 years of age.^23^

The US Preventive Services Task Force updated its fall prevention recommendations in 2024, recommending exercise interventions such as strength and neuromuscular training to reduce injury.^24^ While pickleball offers exercise benefits as well as coordination and balance training, exercises and physical therapy that focuses on gait, balance, functional training, and resistance training are essential to reduce injury during recreational play. Resistance training can improve muscle mass, strength, and physical performance in older adults and is associated with reduced sarcopenia and bone loss.^24^^,^^25^ Further studies are needed to identify interventions for reducing upper extremity injuries in recreational sports especially among elderly women as most of the current literature focuses on rehabilitation and professional sport.

This study's strengths include its use of nationally representative data spanning a decade, addressing limitations of prior small, convenience sample analyses in the existing literature.^8^^,^^23^ Additionally, these results focus on injuries affecting the upper extremity, providing specific insights relevant to hand and upper extremity surgeons. Limitations include the absence of detailed clinical information related to each case's diagnosis and long-term outcome, which prevents the exclusion of repeat visits for the same patient in the analysis. Information about specific diagnoses such as “distal radius fracture” were limited preventing further subgroup analysis. Data from outpatient and telehealth visits, urgent care encounters, and direct admissions to the hospital are not captured in the NEISS database underestimating general incidence of the studied injuries. Injuries that were not as severe (eg, skin tear) may not prompt evaluation by a medical professional creating a bias towards more serious injuries such as fractures. The NEISS may have errors in coding or narrative description affecting case selection. No records of each patient's prior medical history were available to assess for risk factors.

As pickleball continues to grow in popularity, hand surgeons should anticipate increasing clinical demand for management of these injuries. The rise in pickleball-related upper extremity fractures strain emergency services and rehabilitation resources, necessitating public health interventions to prevent injury.^5^^,^^26^ Efforts to mitigate this trend should focus on community education, promotion of fall-prevention strategies, and development of sport-specific protective equipment and rehabilitation protocols. Collaboration between primary care providers and hand specialists can help ensure patients with predisposing factors such as nutritional deficiencies, baseline arthropathy, and frailty avoid serious injury when participating in pickleball. Providing players—particularly older adults—with resources on safe play (e.g., limiting sessions to 1 h, incorporating rest days, and maintaining bone health through regular primary care follow-up) may help reduce injury risk. Early engagement of hand specialists in prevention and outreach can reduce injury burden while supporting the safe enjoyment of this rapidly growing sport.^27^^,^^28^

## 5 Conclusions

Pickleball-related upper extremity injuries have increased substantially over the past decade, with a disproportionate burden among older adults and women and a predominance of wrist fractures. These findings highlight a growing source of demand on emergency and hand surgery services. As participation continues to expand, targeted prevention strategies—including patient education, fall-prevention initiatives, and bone health optimization—will be essential to mitigate injury risk. Greater involvement of hand surgeons in community outreach, care pathway development, and advocacy for protective measures may help reduce morbidity while supporting the safe growth of the sport.

## Funding sources

NA.

## Disclosures

The authors have no disclosure related to the submitted work.

## Presentations

This work has not been presented at the time of submission.

## IRB approval status

According to the Vanderbilt University IRB determination, this secondary analysis of the National Electronic Injury Surveillance System public use data does not meet the definition of human subjects research and therefore did not require additional IRB review. The determination form is available upon request.

## Declaration of competing interest

The authors declare that they have no known competing financial interests or personal relationships that could have appeared to influence the work reported in this paper.
